# Loss of Gap Junction Factor Connexin 43 Results in an Increase of Exosomal Tetraspanins in Human Lung Cancer Cell Line A549

**DOI:** 10.17912/micropub.biology.000683

**Published:** 2022-11-13

**Authors:** Alexandra P Fleck, Ariel B Flotte, Edward P Si, David Mu

**Affiliations:** 1 Eastern Virginia Medical School

## Abstract

Gap junctions (GJs) and small extracellular vesicles such as exosomes are two fundamental intercellular communication (IC) mechanisms. We tested the hypothesis that the two IC mechanisms are connected by gene editing to inactivate a ubiquitously expression GJ factor (i.e., Cx43) in the human lung cancer cell line A549. Surprisingly, we observed that loss of Cx43 led to a buildup of exosomal tetraspanin proteins such as CD63 and CD9. Given the known activities of tetraspanins in cell-cell adhesion and vesicle uptake, our observation establishes an impetus to investigate further how these two IC mechanisms are intertwined

**Figure 1. Loss of Cx43 increases the exosomal CD9 and CD63.**
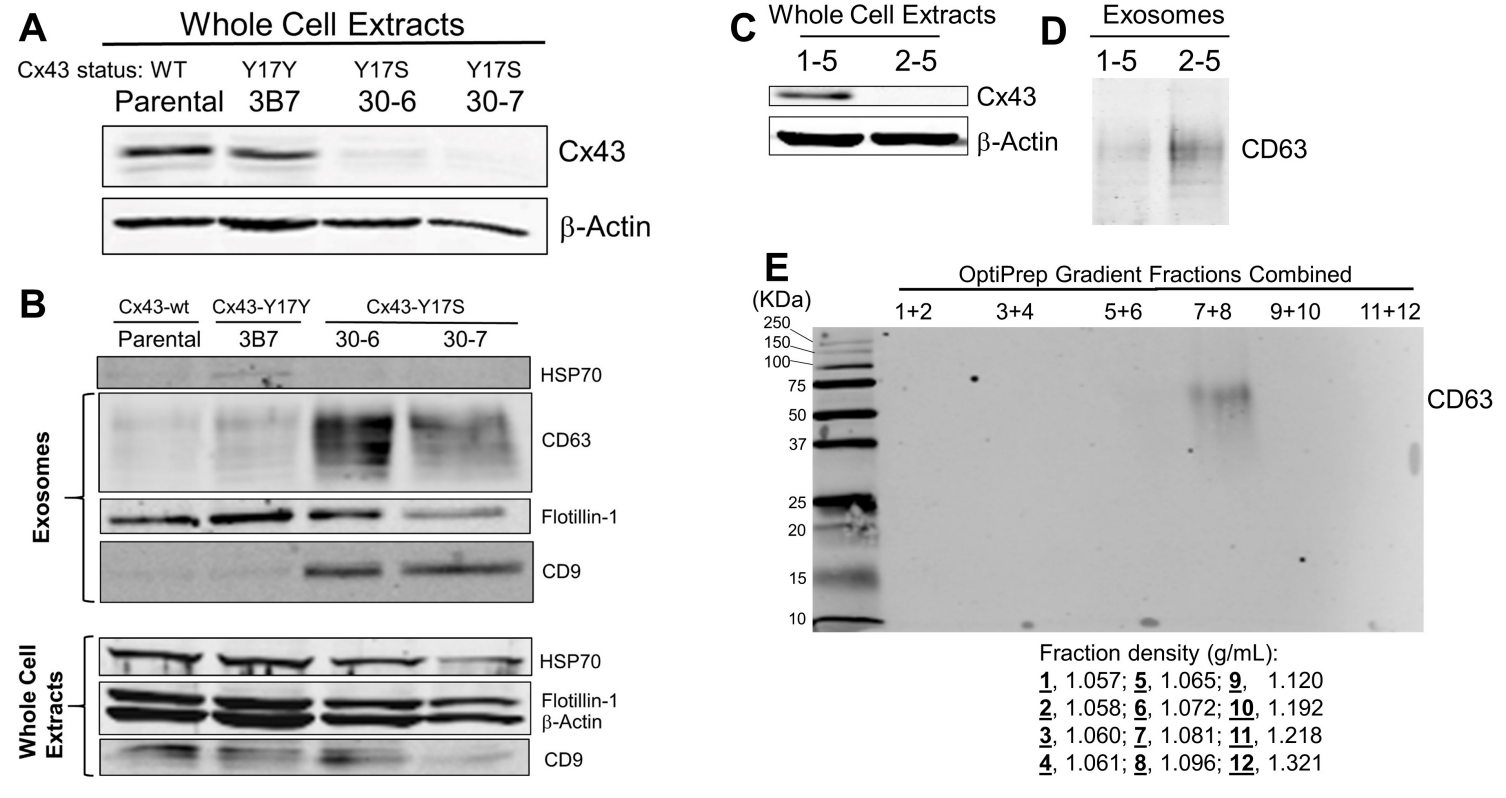
**(A)** Immunoblotting of the whole cell extracts of A549-based cell clones manipulated by the CRISPR technology to knock in either the Y17S mutation or synonymous base alterations (Y17Y) at the Cx43 alleles. “Parental” indicates the extracts derived from the A549 cells that were not CRISPR-treated. **(B)** Immunoblots of exosomes and whole cell extracts derived from the A549 cells were analyzed in A. **(C)** Immunoblotting of the whole cell extracts of an A549 control cell clone (1-5) and a Cx43-KO clone (2-5). **(D)** Immunoblotting of the exosomes secreted by an A549 control cell clone (1-5) and a Cx43-KO clone (2-5). **(E)** Immunoblotting of the fractions for CD63 positivity following an OptiPrep gradient to further resolve the exosomes secreted by Cx43-KO cells. Fractions were combined for gel electrophoresis, and the density of individual fractions is indicated below the immunoblot.

## Description

Gap junctions (GJs) allow the exchange of ions, second messengers, and small metabolites that carry communication signals between cells. An entire GJ channel consists of two hemichannels, each contributed by one of the opposing cells, (Lampe & Laird, 2022; Mese et al., 2007). These hemichannels are hexamers of protein subunits called connexin (Cx). Twenty-one Cx isoforms exist in the human genome, forming GJs between neighboring cells in tissues and organs and playing a role in organismal normal development and function. Extracellular vesicles (EVs) are another means for intercellular communication (IC). EVs are lipid-bound vesicles secreted by cells (Kowal et al., 2014). EVs participate in IC by delivering cargo (e.g., proteins, nucleic acids, or metabolites) from donor to recipient cells (Maas et al., 2017). EVs have three main subtypes: microvesicles, apoptotic bodies, and exosomes. Initially, exosomes were considered a source of cellular dumping. However, we now know that exosomes play an active role in IC, cell maintenance, tumor progression, and even cell immunity (Tkach & Thery, 2016). We hypothesize that the two IC mechanisms, i.e., GJs, and exosomes, are not operating in isolation without regard to each other. To test this hypothesis, we perturbed GJs by editing one of the most studied and ubiquitously expressed Cxs, i.e., Cx43 (Palatinus et al., 2012; Swartzendruber et al., 2020), using the CRISPR technology. Specifically, we used CRISPR to edit the Cx43-Y17 alleles, resulting in the Y17Y synonymous mutation control or the Y17S point mutation in the wild-type (wt) Cx43 alleles of the A549 human lung cancer cell line. We chose the Cx43-Y17S mutation because of its association with oculodentodigital dysplasia (ODDD) (Paznekas et al., 2003). Hereditary Cx43 mutations cause ODDD with phenotypic abnormalities of the eyes, dentition, and digits of the hands and feet (Laird & Lampe, 2022). There are over 70 Cx43 mutations found in ODDD patients, including Y17S. Furthermore, the Y17S mutation has been shown to abolish GJ-dependent IC (Shibayama et al., 2005). By immunoblotting, the Y17S mutation appeared to destabilize the Cx43 protein in two independent cell clones (30-6 and 30-7, Fig 1A) that were verified by Sanger sequencing to contain the Y17S mutation. The level of the Cx43 protein in the 3B7 (Cx43-Y17Y) control cell clone containing sequencing-verified DNA mutations around Y17 but no amino acid changes was comparable to that seen in the parental A549 cells not manipulated by CRISPR (Fig 1A). Next, we isolated the exosomes from the culture media of these cells using the classic differential ultracentrifugation method (Phelps et al., 2019). We authenticated the resultant exosomes using commonly used exosomal markers, including HSP70, CD63, CD9, and Flotillin-1. Unexpectedly, both tetraspanins (CD9 and CD63) were more enriched in the exosomes derived from the Cx43-Y17S cell clones (30-6 and 30-7, Fig 1B). However, HSP70 and Flotillin-1 did not show such a preferential enrichment (Fig 1B). The cell-associated CD63 protein was not detectable by immunoblotting in the whole-cell extracts of the four cell clones (parental, 3B7, 30-6, and 30-7). The apparent instability of the Cx43-Y17S mutant protein prompted us to posit that the loss of the Cx43-Y17S mutant protein (rather than a gain-of-function associated with the Cx43-Y17S mutant protein) in the 30-6 and 30-7 cell clones was responsible for the increases of exosomal CD63 and CD9. To test this hypothesis, we applied a lentiviral CRISPR vector targeting the Cx43-Y17 alleles in A549 cells without a DNA repair template oligonucleotide to create a Cx43-knockout (Cx43-KO) cell clones. A Cx43-KO cell clone, termed 2-5, was verified by Sanger DNA sequencing to contain a single-base insertion near Y17 and predicted to produce a truncated Cx43 mutant protein of 41 amino acids in length. Cell clone 1-5 is a control cell clone treated with the lentiviral CRISPR vector lacking any guide RNA. Immunoblots of the whole-cell extracts of 1-5 and 2-5 confirmed the loss of full-length Cx43 protein in 2-5 (Fig 1C). Intriguingly, immunoblotting of the exosomes secreted by 1-5 and 2-5 cells revealed an increased CD63 presence in the exosomes of 2-5 cells compared with the exosomes of the 1-5 control cells (Fig 1D). This observation was in line with the notion that loss of Cx43 would increase exosomal tetraspanins. To determine if the surged CD63 was associated with exosomes (not as a separate entity being copurified with exosomes), we performed a discontinuous OptiPrep density gradient to fractionate the differential ultracentrifugation-derived exosomes of Cx43-KO cells. To ensure a sufficient protein loading in each electrophoresis lane for optimal immune detection, we coupled up the 12 fractions generated by the discontinuous OptiPrep density gradient. The only combined fractions positive for CD63 were detected in fractions 7 and 8 of the density range (1.080 – 1.096 g/mL), which coincided with the known exosomal densities of 1.08 – 1.19 g/mL) (Greening et al., 2015). In this study, we did not detect obvious morphological alterations in Cx43-KO cells. In future studies, we will monitor cellular morphological changes following any Cx43 perturbation.

## Methods

**Cell Lines**

A549 cells were from ATCC (#CCL-185) and maintained in RPMI-1640 medium (10-041-CV, Corning Life Sciences) supplemented with 10% fetal bovine serum (FBS) and 1X penicillin/streptomycin. Experimental findings and data presented were representative experiments from a set of three replicates.

**CRISPR-Based Gene Editing**

Cx43 gene editing to Cx43-Y17Y or Cx43-Y17S: Cx43G3 (CACCGGTTCAAGCCTACTCAAC

TGC) and Cx43G4 (AAACGCAGTTGAGTAGGCTTGAACC) oligonucleotides were annealed and ligated into HF-PX459-V2 (Addgene, #118632) vector cut by BbsI. Two DNA repair template oligonucleotide: CX43HDR1 (for the Cx43-Y17Y genotype): AGGCAACATGGGTGACTG

GAGCGCCTTAGGCAAACTCCTTGACAAGGTTCAAGCTTATTCTACAGCTGGAGGGAAGGTGTGGCTGTCAGTACTTTTCATTTTCCGAATCCTGCTGCTGGGGA. CX43HDR2 (for the Cx43-Y17S genotype): AGGCAACATGGGTGACTGGAGCGCCTTAGGCAAACTCCTTGACAAGGTT

CAAGCTTCTTCTACAGCTGGAGGGAAGGTGTGGCTGTCAGTACTTTTCATTTTCCGAATCCTGCTGCTGGGGA. The Cx43-Y17-targeting HF-PX459-V2 vector (i.e., Cx43G3/4-HF-PX459-V2) was cotransfected with either CX43HDR1 or CX43HDR2 (vector: DNA repair template oligo = 5: 1 (w/w)) into A549 cells using Lipofectamine 3000 (Thermo Fisher). After a 4-day puromycin treatment (1 μg/mL) to eliminate untransfected cells, clonal cells were isolated by limiting dilution. Cx43-KO cell clones : Annealed Cx43G3 and Cx43G4 oligonucleotides were ligated into BsmBI-cut Lenti-CRISPR-V2 plasmid (Addgene, #52961), resulting in Cx43G3/4-lenti-CRISPR-V2 plasmid. To produce lentiviruses, 293T cells cultured in 100mm dishes were transfected using the calcium phosphate method with a mixture of plasmids of this combination (10μg Lenti-CRISPR-V2 + 7.5μg psPAX2 (Addgene, #12260) + 2.5μg pCMV-VSV-G (Addgene, #8454)). A549 cells infected with lentiviruses were puromycin selected at 1 μg/mL for 4 ~ 5 days to eliminate uninfected cells. Clonal cells were obtained by limiting dilution.

**Exosome Isolation and Characterization**

All exosomes were isolated from cell culture supernatants of cells close to 95% confluency using a differential ultracentrifugation (Phelps et al., 2019). For exosome preparation, A549 cells were cultured in 150 mm dishes with 30 mL of Opti-MEM media (31985-070, Thermo Fisher) supplemented with 1% of exosome-depleted FBS for 24-72 hours. The cell culture supernatant was collected and sequentially centrifuged at 500 g for 10 minutes, 2000 g for 20 minutes, 20,000 g for 30 minutes, and 100,000 g for 90 minutes to obtain a pellet of exosomes. The pellet was suspended in phosphate-buffered saline (PBS, 21-040-CV, Corning Life Sciences) and stored at -80 degrees Celsius. The size scale and concentration of exosomes were measured using a nanoparticle tracking analysis (NTA) device, NanoSight NS300 (Malvern Panalytical). According to the manufacturer's instructions, we determined the protein concentration of the isolated exosomes using a micro BCA protein assay kit (23235, Thermo Scientific). A standard curve was derived with nine serial dilution points of bovine serum albumin (BSA) standards and a working reagent.

**Discontinuous OptiPrep Density Gradient**

A discontinuous OptiPrep (60% iodixanol in water, 1114542, Axis-shield) density gradient was used to purify the isolated exosomes further. Solutions of 5, 10, 20, and 40% iodixanol were made by mixing a homogenization medium (0.25 M sucrose, 1mM EDTA, 10 mM HEPES-NaOH, pH 7.4) with a 40% iodixanol working solution. The working solution incorporated combining a working solution buffer (0.25 M sucrose, 3 mM EDTA, 30 mM HEPES-NaOH, pH 7.4) and a stock solution of OptiPrep (60% (w/v) aqueous iodixanol solution). The gradient was formed by layering 3.0 mL of 40%, 2.5 mL of 20%, 2.5 mL of 10%, and 2.0 mL of 5% in a 14 mL open-top polyallomer tube (331374, Beckman Coulter). The pellet was suspended in 1 mL of homogenization medium twice, loaded onto the top of the gradient, and then centrifuged at 200,000 g and 4 degrees Celsius for 18 hours. Gradient fractions of 1 mL were collected from the top of the gradient, generating 12 fractions. After obtaining the refractive index of each fraction (Fisher refractometer), the fractions were diluted with 3 mL of PBS and centrifuged for 2 hours at 200,000 g and 4 degrees Celsius. The pellets were resuspended in 25 uL of PBS twice. A standard curve was made using the manufacturer's absorbance values of 6-50% iodixanol-sucrose solutions to estimate each fraction's density.

**Immunoblotting**

The proteins were separated using a 12% polyacrylamide gel and transferred to a nitrocellulose membrane. The primary antibodies used for immunoblotting included: anti-CD63 (H5C6, Developmental Studies Hybridoma Bank) mouse monoclonal antibodies raised against full-length human CD63 at 1:1000 dilution, anti-CD9 (D801A, Cell Signaling Technology) rabbit monoclonal antibodies raised against full-length human CD9 at 1:1000 dilution, anti-Flotillin-1 (D2V7J, Cell Signaling Technology) rabbit monoclonal antibodies raised against full-length human Flotillin-1 at 1:1000 dilution, anti-β-actin (8H10D10, Cell Signaling Technology) mouse monoclonal antibodies raised against full-length human β-actin at 1:500 dilution, and anti-Cx43 (3512S, Cell Signaling Technology) rabbit monoclonal antibodies raised against full-length human Cx43 at 1:1000 dilution. Secondary antibodies (926-68070 and 926-32211, Licor) were used and read on a Licor Odyssey Infrared Imaging System 9120 scanner. Whole-cell extracts are made by lysing PBS-washed cells on culture dishes with 1x Laemmli sample buffer at 500mL per 100mm dish. The resultant lysates were passed through 25-gauge needles to shear genomic DNA and reduce extract viscosity.

**Photo Documentation**

Pictures were taken with a Licor Odyssey Infrared Imaging System 9120 scanner. Adobe Photoshop 7.0 was used for cropping and careful brightness adjustments. All figures were arranged using Microsoft PowerPoint 2016.
